# Influence of low FODMAP-gluten free diet on gut microbiota alterations and symptom severity in Iranian patients with irritable bowel syndrome

**DOI:** 10.1186/s12876-021-01868-5

**Published:** 2021-07-14

**Authors:** Kaveh Naseri, Hossein Dabiri, Mohammad Rostami-Nejad, Abbas Yadegar, Hamidreza Houri, Meysam Olfatifar, Amir Sadeghi, Saeede Saadati, Carolina Ciacci, Paola Iovino, Mohammad Reza Zali

**Affiliations:** 1 Department of Microbiology, School of Medicine, Shahid Beheshti University of Medical Sciences, Tehran, Iran; 2 Celiac Disease Department, Gastroenterology and Liver Diseases Research Center, Research Institute for Gastroenterology and Liver Diseases, Shahid Beheshti University of Medical Sciences, Tehran, Iran; 3 Foodborne and Waterborne Diseases Research Center, Research Institute for Gastroenterology and Liver Diseases, Shahid Beheshti University of Medical Sciences, Shahid Arabi Ave., Yemen St., Velenjak, Tehran, Iran; 4 Gastrointestinal Unit, Department of Medicine, Surgery and Dentistry Scuola Medica Salernitana, Università di Salerno, Via Allende, 84081 Salerno, Italy

**Keywords:** Irritable bowel syndrome, Gluten-free diet, Low-FODMAP, Gut microbiota, IBS-SSS, Iran

## Abstract

**Background and objective:**

Recently, dietary restriction of fermentable carbohydrates (a low-FODMAP diet) in combination with a gluten-free diet (GFD) has been proposed to reduce the symptoms in irritable bowel syndrome (IBS) patients. Different studies reported that IBS has been associated with dysbiosis in the gut microbiota. Additionally, a few studies have reported inflammation in the gastrointestinal (GI) system of adults with IBS. In this study, we aimed to investigate the effects of low FODMAP-gluten free diet (LF-GFD) on clinical symptoms, intestinal microbiota diversity, and fecal calprotectin (FC) level in Iranian patients with IBS.

**Design:**

In this clinical trial study, 42 patients with IBS (Rome IV criteria) underwent LF-GFD intervention for 6 weeks. Symptoms were assessed using the IBS symptom severity scoring (IBS-SSS), and fecal samples were collected at baseline and after intervention and analyzed by quantitative 16 S rRNA PCR assay. The diversity of gut microbiota compared before and after 6 weeks of dietary intervention. FC was also analyzed by the ELISA method.

**Results:**

Thirty patients (mean age 37.8 ± 10.7 years) completed the 6-week diet. The IBS-SSS was significantly (*P* = 0.001) reduced after LF-GFD intervention compared to the baseline. Significant microbial differences before and after intervention were noticed in fecal samples. A significant increase was found in *Bacteroidetes*, and the *Firmicutes* to *Bacteroidetes* (F/B) ratio was significantly (*P* = 0.001) decreased after the dietary intervention. The value of FC was significantly decreased after 6 weeks of dietary intervention (*P* = 0.001).

**Conclusions:**

Our study suggests that patients with IBS under an LF-GFD had a significant improvement in IBS symptoms severity, with reduced FC level following normalization of their gut microbiota composition. Further rigorous trials are needed to establish a long-term efficacy and safety of this dietary intervention for personalized nutrition in IBS.

Clinical Trial Registry Number: IRCT20100524004010N26.

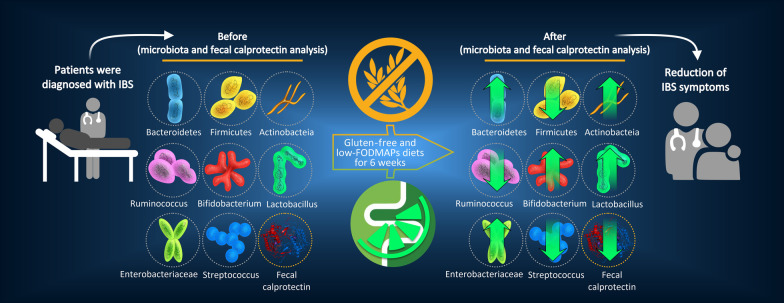

**Supplementary Information:**

The online version contains supplementary material available at 10.1186/s12876-021-01868-5.

## Introduction

Irritable bowel syndrome (IBS) is one of the most commonly diagnosed functional gastrointestinal disorders accounting for around 10% of the world’s population [1]. This functional bowel disorder can be classified into different categories: diarrhea-predominant (IBS-D), constipation-predominant (IBS-C), mixed (IBS-M), and unclassified (IBS-U) [2].

The pathogenesis of IBS is complex and not yet clearly defined, but numerous pathophysiological mechanisms have been proposed including brain-gut dysfunction, intestinal dysmotility, visceral hypersensitivity, inflammation, psychosocial stressors, altered levels of gastrointestinal neuropeptides and hormones, and particularly imbalance in the composition of gut microbiota, called “microbiome dysbiosis” [3, 4]. It is now well evident that microbiome dysbiosis-related disorders and IBS have been reported to have similar clinical features, and more recently, IBS has been found to be in association with the bacterial community shift in the large intestine [5]. Furthermore, restoration and modulation of gut microbiota through consumption of probiotics, prebiotics, and symbiotic, which result in improvement of the IBS-related symptoms highlights the important role of gut microbiota dysbiosis in the pathogenesis of IBS [6, 7].

Normal gut microbiome inhibits the overgrowth and colonization of pathogenic organisms by competition for nutrients and attachment sites in the gut epithelium, production of antibacterial substances, as well as enhancement of the host immune responses [8, 9]. Several previous studies have described that alterations in the gut microbiota such as increased number of *Enterobacteriaceae* and *Bacteroidetes* or decreased number of beneficial organisms like *Bifidobacterium* and *Lactobacillaceae*, can trigger a gut immune response, impair gastrointestinal functions and enhance disease susceptibility [6, 10].

A majority of IBS patients suffer from abdominal discomfort or pain and bloating after ingestion of certain food items that may contain IBS-associated triggers [11, 12]. In addition, a number of studies suggest that environmental factors such as diet, lifestyle, and antibiotics and medications exert a significant impact on the gut microbiome [13]. Recently, dietary components including wheat, gluten, and fermentable oligo-di-mono-saccharides and polyols (FODMAP) have been suggested to play an essential role in the induction of IBS symptoms [14]. Accordingly, there is some evidence supporting a clinically relevant positive effect for low-FODMAP and gluten-free diets in patients with IBS [15].

The significant effect acknowledged to date is alterations in gut microbiota by varying gluten intake, such as altering total microbiota abundance and changing the relative amount of *Bifidobacteria* [12, 16]. However, the roles of alterations of gut microbiota due to the reduction in FODMAP intake in ongoing efficacy have yet to be explored. Herein, we aimed to investigate the impact of a 6-week LF-GFD on gut microbiota alterations in Iranian patients with IBS. Additionally, in order to determine the inflammation status of patients, we examined the level of fecal calprotectin (FC) before and after dietary intervention.

## Materials and methods

### Study population

**Fig. 1:**
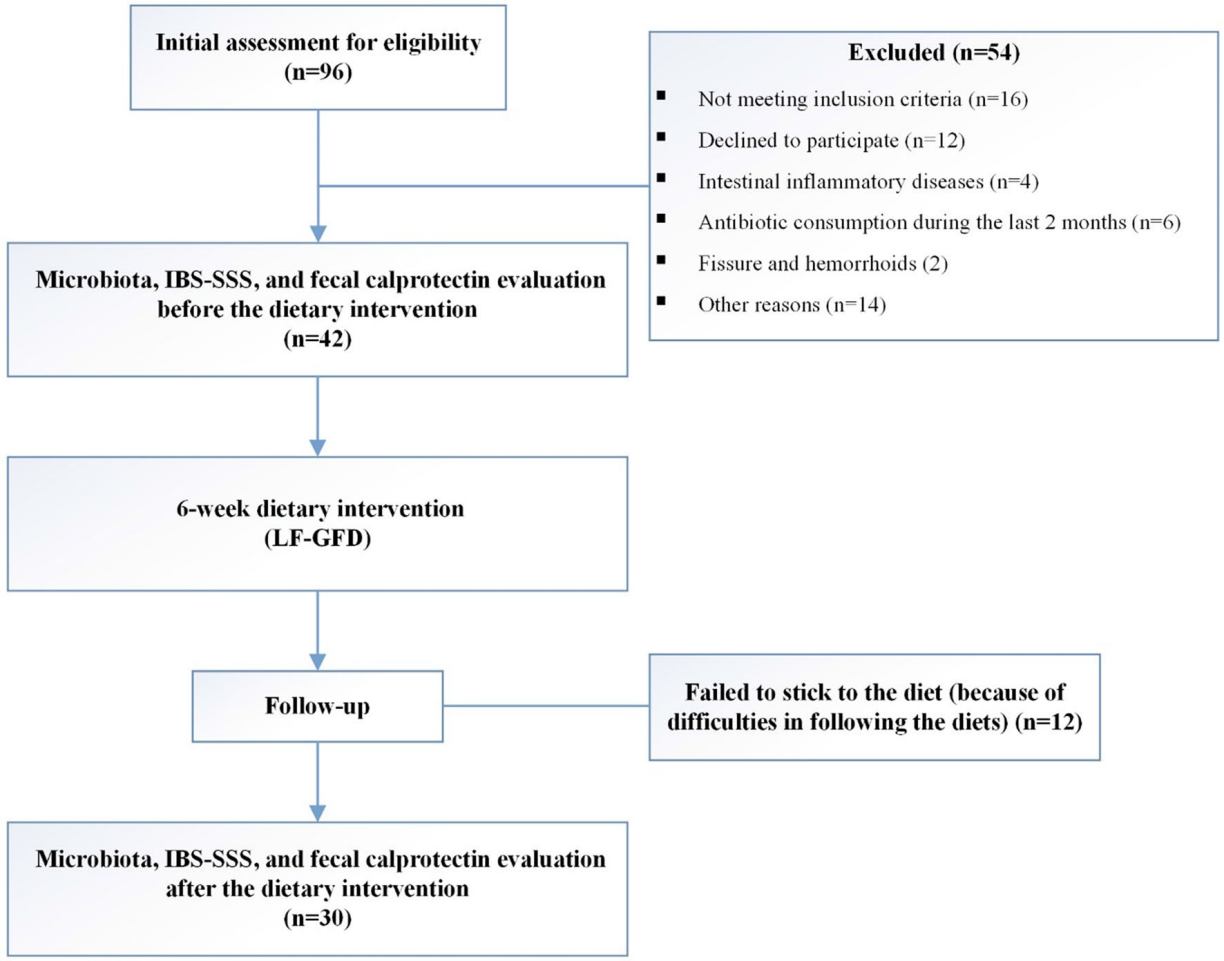
Schematic of recruitment process and study design

Ninety-six consecutive IBS patients were screened for eligibility; 54 patients did not meet the initial assessment criteria, and 12 patients discontinued the dietary intervention during the follow-up phase and 30 patients completed the study. A flowchart of the recruitment process and study design is shown in Fig. 1. Forty-two eligible patients with IBS, aged 18–59 years (mean age 37.8 ± 10.7 years), and had a physician diagnosis of IBS according to the Rome IV diagnostic criteria, and did not have any other gastrointestinal disorders were recruited in this study [17]. Patients with the following criteria were excluded: patients with a history of celiac disease (CD), inflammatory bowel disease (IBD), liver diseases, gastrointestinal surgery, cancer, use of non-steroidal anti-inflammatory drugs (NSAIDs), excessive alcohol consumption, systemic use of immunosuppressive agents, and poorly controlled psychiatric disease. In addition, colonoscopy and biopsy specimens were performed in order to include patients with normal mucosa and excluding those with fissures, hemorrhoids, and microscopic colitis. Moreover, those who used broad-spectrum antibiotics, probiotics, or any other drugs that affect the bowel function within the last 4 weeks prior to the study were also excluded. Active participation in another form of dietary therapy at the time of enrollment (i.e., low carbohydrate, high protein) was not considered in this study.

### Study design and procedures

This study was an uncontrolled, open-label clinical trial study, which was conducted in the Research Institute for Gastroenterology and Liver Diseases (RIGLD) at Taleghani Hospital in Tehran, Iran. All the participants were recruited between March 2018 and August 2019. This was a dietary trial and the dietitian was not blinded. Patients were referred to the trained research dietitian at the RIGLD and were counseled on their allocated diets. A standardized questionnaire including demographic information, usual diet, medication history, and underlying health condition and clinical symptoms was recorded for all patients. All IBS patients fulfilling inclusion criteria were counseled by the dietitian and given an LF-GFD for a 6-week run-in period. Fecal samples were collected from all patients before and after the completion of the study period for microbiota analysis. In order to assess the effect of the dietary interventions and the severity of IBS symptoms, the IBS symptom severity score (IBS-SSS) was applied [18]. The overall IBS-SSS ranges from 0 to 500, and all patients were divided into 3 severity groups by use of the accepted cut-off values: < 175, mild IBS; 175–300, moderate IBS; > 300, severe IBS. A higher score implicates more severe symptoms. Patients also classified into the following categories: (1) Defecation, (2) Changes in frequency, and (3) Changes in form of stool.

The study protocol was approved by the Ethical Review Committee of RIGLD at Shahid Beheshti University of Medical Sciences (Project No. IR.SBMU.RIGLD.REC.1396.154). The study was performed according to the revised Declaration of Helsinki 2013 [19] and informed consents were obtained from all subjects and/or their legal guardians prior to sample collection. This study was registered in the Iranian Registry of Clinical Trials on 20/10/2018 and the clinical trial registration number is IRCT20100524004010N26.

### Collection of fecal samples

Fresh stool samples were collected from every subject enrolled in this study at baseline and after 6 weeks following dietary therapy. All samples were homogenized through agitation using a vortex and divided into three aliquots within 3 h of defecation. The aliquots were immediately frozen and stored at − 80 °C in screw-capped cryovial tubes until used for DNA extraction.

### DNA extraction from fecal samples

Total DNA content was extracted from stool samples using the QIAamp DNA Stool Mini Kit (Qiagen Retsch GmbH, Hannover, Germany) according to the manufacturer’s instructions with some modifications. DNA concentration was quantified by NanoDrop ND-2000 Spectrophotometer (NanoDrop products, Wilmington, DE, USA). The concentration and purity of extracted DNA were assessed by Nanodrop (DeNovix Inc., USA). Extracted DNA samples were stored at − 20 °C until further analysis.

### Diet

The main aim of the nutritional assessment was the evaluation of participants’ nutritional status. After investigation of the recordings of the dietary recall for 3 days (1 weekend and 2 workdays), a personalized LF-GFD adjusted to match energy, macronutrients, and micronutrients daily requirements was designed for each participant. To be precise, if a food is gluten-free, it does not necessarily mean that it will be low in FODMAP contents. In other words, some gluten-containing foods are low FODMAP foods. However, in this study, we planned to apply an intervention that was not only low in FODMAP contents but also was free of gluten. In Additional file 1: Table S1, some examples of high FODMAP gluten-free foods and some examples of gluten-containing low FODMAP foods are described, all of which were eliminated in the LF-GFD. All of the diets were prepared by a trained nutritionist who was responsible for doing nutrition counseling (an average of 1 h), prescribing the diets, and being in contact with patients through telephone and/or email. Compliance with the diet was determined by the evaluation of the 3 days dietary recall by the same nutritionist. At the beginning of the study, an in-depth GFD review, high FODMAP foods, and low FODMAP alternatives were explained to all the participants, in addition to the education of how to modify the FODMAP content towards the LFD, as Iranian dishes have a high level of FODMAP content. An example of one day LF-GFD which should have been followed during the study and a regular diet before start of the study is shown in Table 1.

**Table 1:** Examples of the two different prescribed diets before and during the study for a typical day

Meal	LF-GFD	Regular diet
Breakfast	1 cup of tea 90 g of gluten-free muffin	1 glass of milk 3 slices of bread 3 teaspoons of cherry jam
Morning snack	1 banana	1–3 slices of cookies
Lunch	20 tablespoons of rice 90 g of seafood Vegetable salad (1 cucumber + 1 tomato + 1 cup of lettuce)	20 tablespoon of rice ½ cup of onion ½ cup of beans 60 g of red meat 3 slices of bread
Afternoon snack	1 cup of tea 30 g of gluten-free biscuits	1 cup of Fruit of the season 100 g of ice cream
Dinner	2 slices of whole grain gluten-free bread 60 g of chicken 100 g of carrots	90 g of chicken or regular pasta 100 g of tomato 3 slices of bread 1 cup of regular yogurt
During the day	90 g of grapes	2 cup of Fruit of the season

Dietary data represent the typical diet for a patient with an approximate energy expenditure of 1600 kcal/day

*FODMAP* fermentable, oligosaccharides, disaccharides, monosaccharides, and polyols, *GFD* gluten-free diet, *LFD* low FODMAP diet, *LF-GFD* low FODMAP gluten-free diet

### Microbiota analysis by quantitative real-time PCR (qPCR)

In the current study, a qPCR assay was performed for the enumeration of eight bacterial phyla, families, and genera including *Firmicutes*, *Bacteroidetes*, *Actinobacteria*, *Enterobacteriaceae*, *Bifidobacterium*, *Lactobacillus*, *Ruminococcus*, and *Streptococcus*. The qPCR was carried out by SYBR Green chemistry using universal and group-specific primers based on the bacterial 16 S rRNA sequences presented in Additional file 2: Table S2. Each PCR reaction was performed in a final volume of 25 µL, comprising of 12.5 µL of SYBR green PCR master mix (Ampliqon, Odense, Denmark), 1 µL of 10 pmol of forward and reverse primers, and 100 ng of the DNA template. The reaction parameters for amplification were 95 °C for 10 min and 40 cycles at 95 °C for 20 s, 30 s of annealing at optimal temperature for each primer pair as indicated in Additional file 2: Table S2, and 72 °C for 20 s. All PCR amplifications were carried out in triplicate by using a Rotor-Gene® Q (Qiagen, Germany) real-time PCR system. The accuracy of amplification was determined by melting curve analysis with increasing temperature from 60 to 95 °C (at the regular increment of 0.5 °C for 5 s) to confirm the specificity of amplification. The relative abundance of each taxon before and after the dietary therapy was calculated according to the ratio of 16 S rRNA copy number of specific bacteria to total 16 S rRNA copy number of universal bacteria using the previously described method [20]. Accordingly, the average Ct value obtained from each primer pair was transformed into a percentage using the following formula:

$$X=\frac{{({\text{Eff}}.\; {\text{Univ}})}^{{{\rm Ct}}}\;{\text{univ}}}{{({\text{Eff}}.\; {\text{Spec}})}^{{\rm Ct}} \;{\text{spec}}}\times 100$$

The “Eff. Univ” refers to the calculated efficiency of the universal primers (2 = 100 % and 1 = 0 %) and “Eff. Spec” indicates the efficiency of the taxon-specific primers. “Ct univ” and “Ct spec” represent the threshold cycles registered by the thermocycler. “X” represents the percentage (%) of 16 S taxon-specific copy numbers existing in a sample.

### Fecal calprotectin measurement

Before and after the dietary therapy, first-morning stool samples were collected from patients and stored at − 80 °C after initial storage. FC was measured by using BÜHLMANN fCAL^®^ ELISA kit (Bühlmann Laboratories, Schönenbuch, Switzerland) according to the manufacturer’s instructions. An FC level < 50 µg/g stool was considered to be within the normal range.

### Statistical analysis

Spearman’s correlation analysis was used for non-parametric values and student *t*-test and Mann-Whitney test were used for the analysis of parametric data. The PCA (principle component analysis) plot was drawn by using the FactoMineR and Factoextra packages from the open-source statistical program R version 3.6.1 (R Core Team, Vienna, Austria). The relative abundance of microbiota was graphed using GraphPad Prism software version 8.3.0 (GraphPad Software, San Diego, CA, USA). Differences were considered to be statistically significant when *P < 0.05, **P < 0.01, and ***P < 0.001.

## Results

### Baseline characteristics

Out of 96 IBS patients, 30 patients completed the study period (a six-week of LF-GFD), of whom 15 (50%) patients were male, mean age 35.7 years (range 21–57 years), and 15 patients (50%) were female, mean age 39.8 years (range 18–59 years). Most of the IBS patients were sub-classified into IBS-D according to the Rome IV criteria (Table 2).

**Table 2:** Baseline characteristics of study participants at enrollment

Baseline characteristics	Number (%)
Age (years), mean (range)	37.8 (18–59)
Gender (M/F)	15/15
Smoking, yes (%)	9 (30 %)
IBS-SSS, mean (range)	326 (250–475)
*IBS subtypes*	
IBS-D	16 (54)
IBS-C	10 (33)
IBS-M	3 (10)
IBS-U	1 (3)
*Ethnicity*	
Persian Turk Kurd Lur Others	10 11 4 3 2

### Effects of the GFD + low-FODMAP diet on the microbiota diversity

**Fig. 2:**
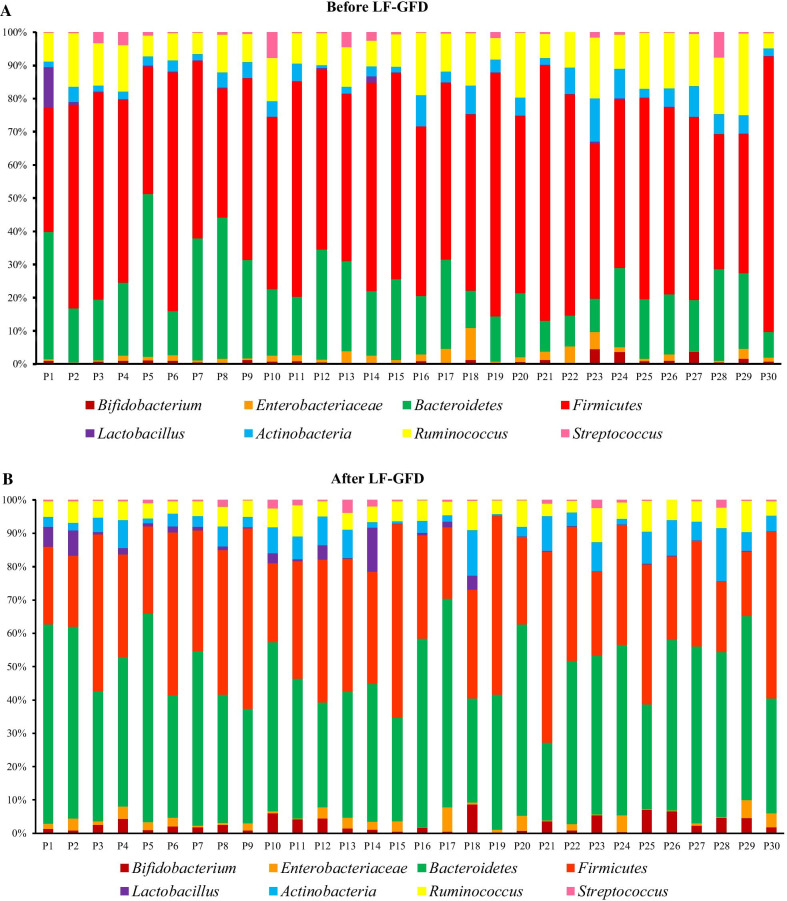
The relative abundance and diversity of intestinal microbiota in IBS patients before and after LF-GFD are illustrated in (**a**) and (**b**), respectively. Each color corresponds to a type of microbiota included in this study

**Fig. 3:**
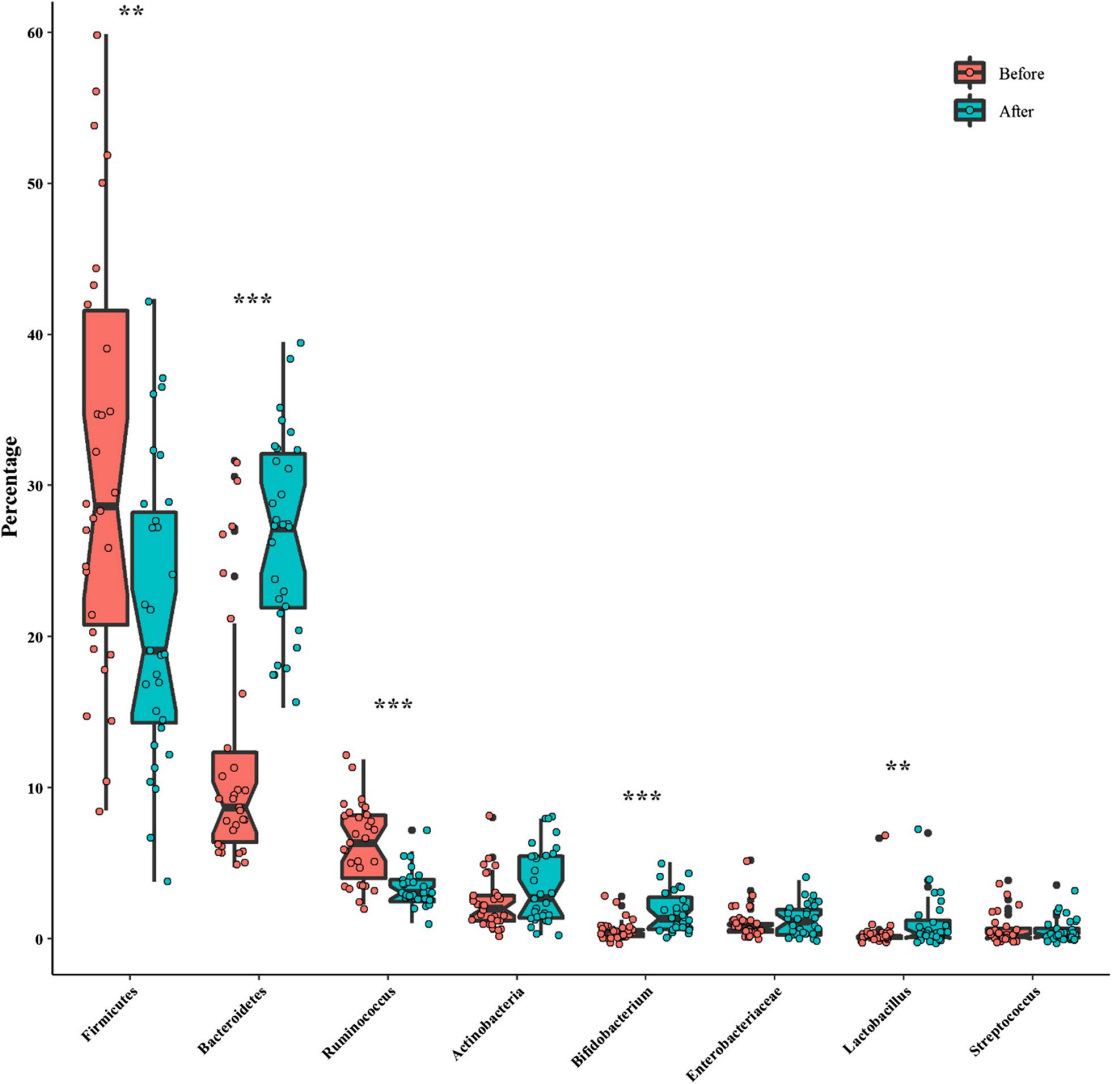
Box plot for the distribution of the selected bacterial taxa by the median abundance that constitutes the fecal microbiota in IBS patients before and after LF-GFD

**Fig. 4:**
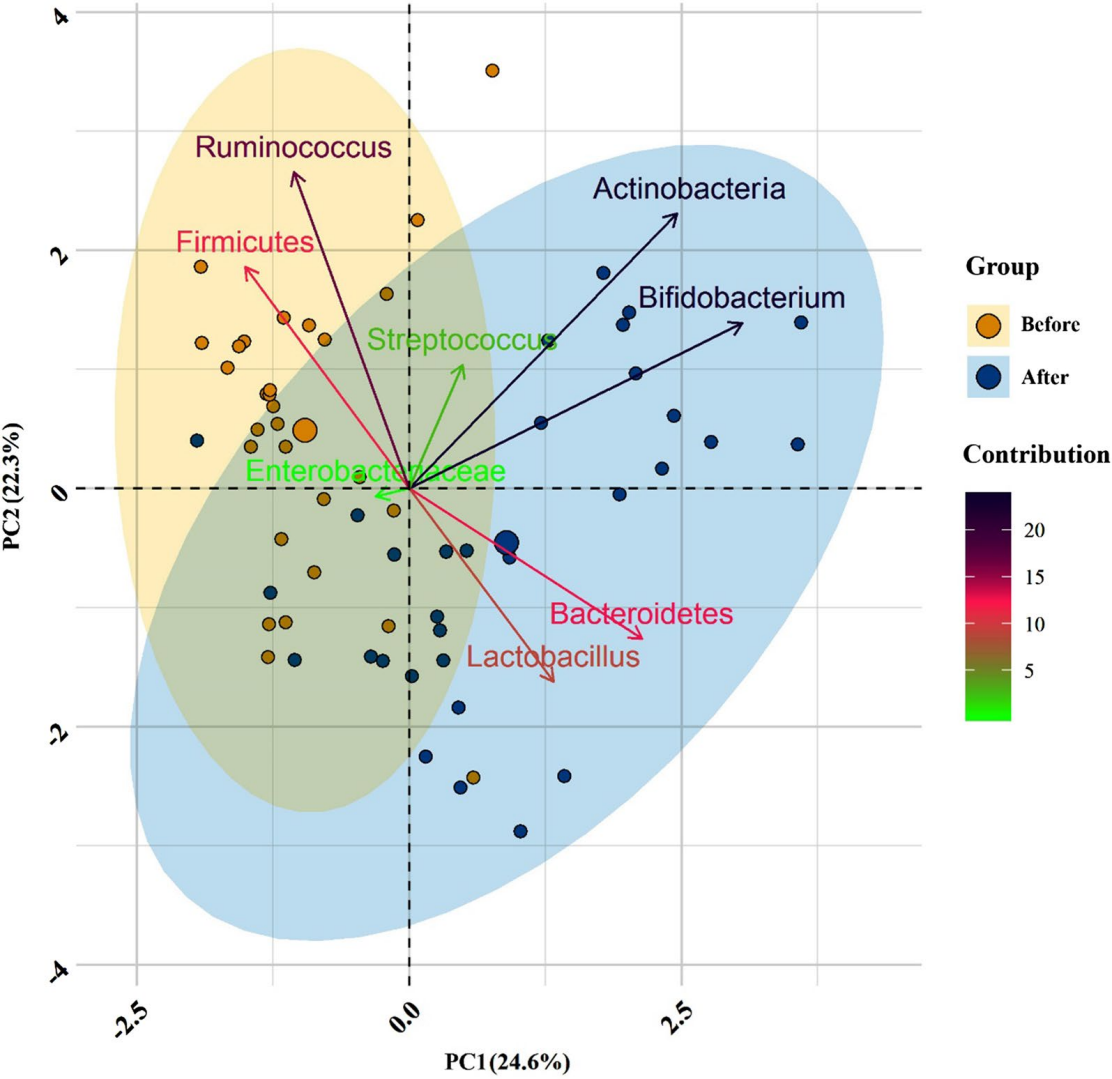
Bacterial community clustering using principal component analysis (PCA) before and after LF-GFD in IBS patients. Percentage values in parentheses next to PC1 and PC2 represent the percentage of variance explained by each component. Arrows show the contribution of each type of microbiota on the PC1 and PC2. Each data point denotes an individual patient, colored based on their group

**Fig. 5:**
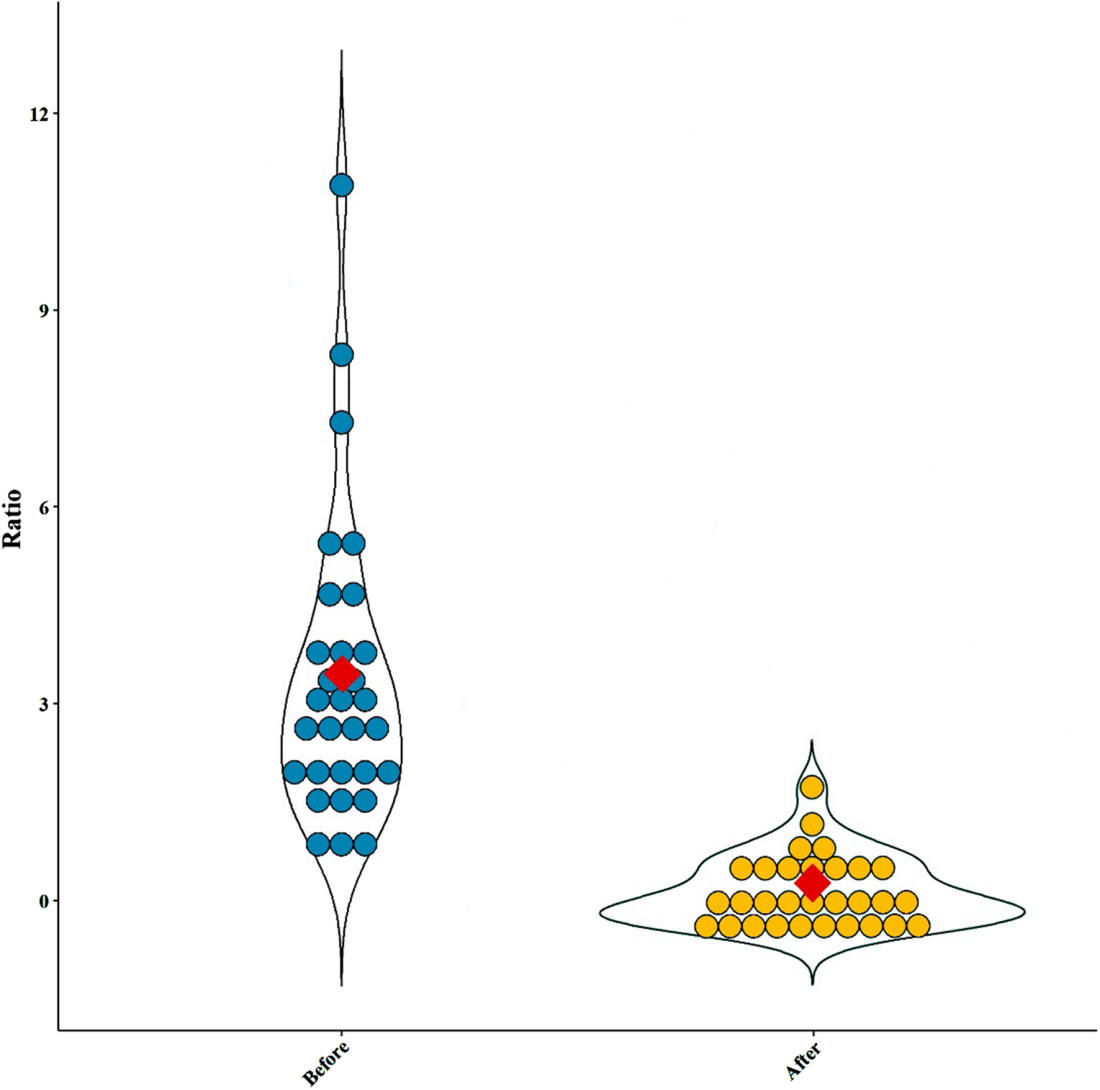
Violin plots showing the Firmicutes to Bacteroidetes (F/B) ratio before and after LF-GFD in IBS patients. This ratio was significantly (*P* = 0.001) decreased after the dietary intervention compared with baseline. Mean values of the F/B ratio are marked as the red rhombus

Our intestinal microbiota analysis was based on qPCR amplification to calculate the ratio of 16 S rRNA copy number of each bacterial taxa before and after the dietary intervention. The relative abundance and diversity of intestinal microbiota among IBS patients before and after the LF-GFD intervention is illustrated in Fig. 2a and b. The predominant phylum in IBS patients before the LF-GFD was *Firmicutes* (31.59 %), which significantly reduced after the dietary intervention (22.17%; *P* = 0.003). *Bacteroidetes* was significantly the most abundant phylum after the dietary intervention, (from 11.69% (baseline) to 26.65%; *P* = 0.001). The relative abundance of phylum *Actinobacteria* was also increased after the dietary intervention, but this difference was not statistically significant (*P* = 0.12). No significant (*P* = 0.63) alterations were also observed in the relative abundance of *Enterobacteriaceae* before and after the intervention. However, the percentage of *Ruminococcaceae* significantly decreased from 6.4 to 3.45% (*P* = 0.001). At the genus level, the relative abundance of *Bifidobacterium* and *Lactobacillus* significantly (*P* = 0.001 and *P* = 0.006, respectively) increased after the intervention, whereas the percentage of *Streptococcus* remained almost similar to its baseline abundance with no significant changes (*P* = 0.72). The mean percentage and distribution of the selected bacterial taxa before and after LF-GFD in IBS patients are illustrated in Fig. 3 and Additional file 3: Figure S1. In addition, principal component analysis (PCA) also revealed that taxonomic profiles were notably different in the microbial communities before and after the dietary intervention among IBS patients as schematically depicted in Fig. 4. The ratio of *Firmicutes* to *Bacteroidetes* (F/B) was significantly decreased (*P* = 0.001) and shifted from 2.6:1 to 0.8:1 before and after the dietary intervention, respectively. The F/B ratio before and after LF-GFD in IBS patients is shown in Fig. 5.

### Effects of the GFD + low-FODMAP diet on IBS symptom severity

**Fig. 6:**
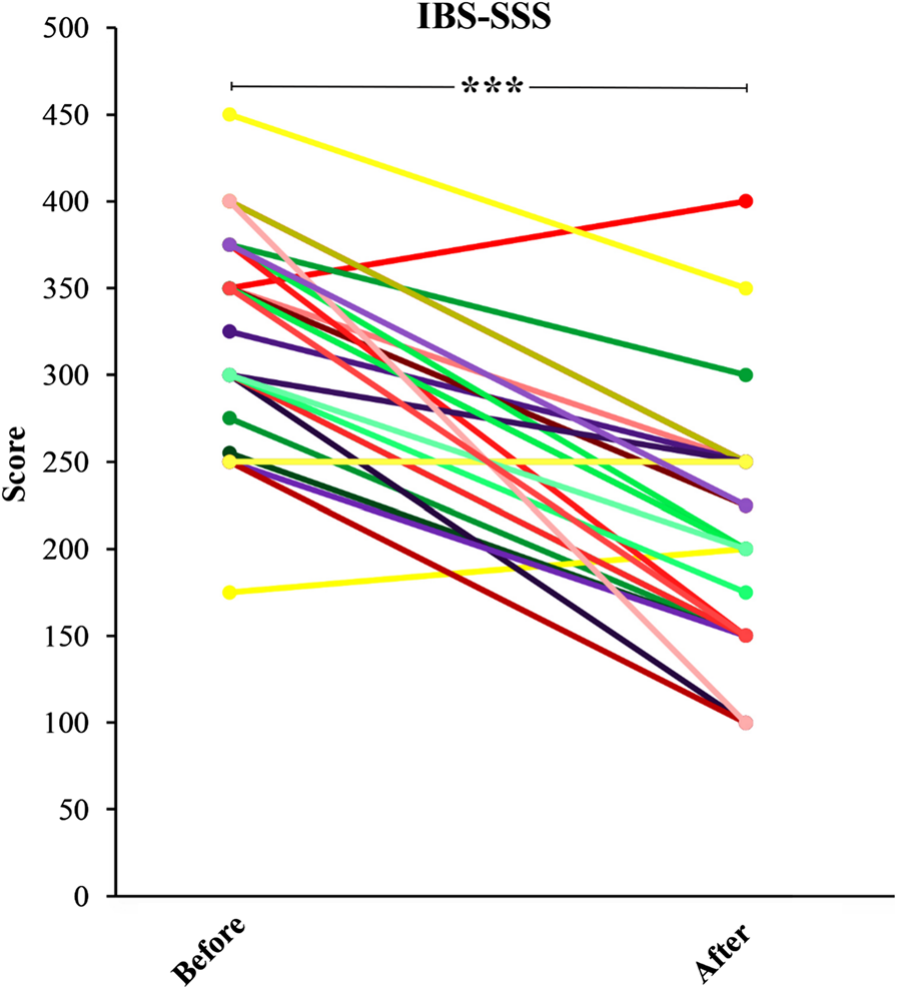
IBS symptom severity score (IBS-SSS) in IBS patients before and after the dietary intervention. IBS-SSS was reduced in patients after the end of the dietary intervention compared with baseline (*P* = 0.001)

Figure 6 represents IBS symptom severity in each patient before and after the dietary intervention. Based on the IBS-SSS, the severity of IBS symptoms was classified as mild (n = 1), moderate (n = 12) and severe (n = 17) at the baseline and before the dietary intervention. After the end of the dietary intervention, the number of patients in each classification changed to mild (n = 8), moderate (n = 20), and severe (n = 2), accordingly (Table 3. Totally, IBS-SSS decreased in 22/30 (73.3%) patients after the dietary intervention compared to the baseline, and this clinical improvement was statistically significant (*P* = 0.001). Approximately, 53  of patients after the end of the dietary intervention experienced a 30–60% reduction in IBS-SSS, while only 3.3% of patients of experienced more than 60% reduction (Table 4).

**Table 3:** Severity scores for mild, moderate and severe IBS patients before and after the dietary intervention

IBS-SSS	Before			IBS-SSS	After		
	Mean (± SD)	Median	Range		Mean (± SD)	Median	Range
Mild (n = 1)	175 (-)	175	–	Mild (n = 8)	146.8 (± 19.5)	150	100–175
Moderate (n = 12)	277.5 (± 23.4)	287.5	250–300	Moderate (n = 20)	231.25 (± 28.6)	237.5	200–300
Severe (n = 17)	369.1 (± 29.1)	350	300–450	Severe (n = 2)	375 (± 25)	375	350–400

**Table 4:** Frequency distribution of IBS-SSS reduction

IBS-SSS reduction category	After dietary intervention n (%)	*P* value
Decreased/Increased/unchanged	22 (73.3)/2 (6.6)/6 (20)	> 0.001
< 30% reduction	5 (16.6)	
30–60% reduction	16 (53.3)	
> 60% reduction	1 (3.3)	

## Fecal calprotectin

**Fig. 7:**
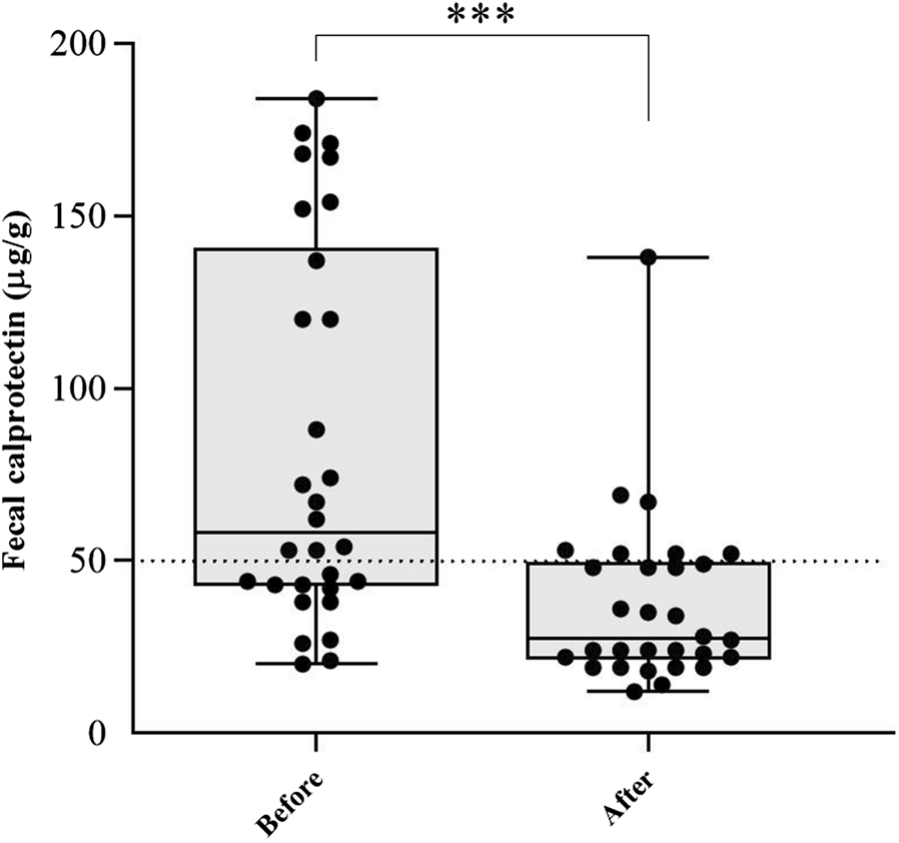
Values of fecal calprotectin level in IBS patients before and after a six-week of LF-GFD. ***P < 0.001

The mean FC values in the IBS patients at the baseline and after a 6-week LF-GFD was shown in Fig. 7. The value of FC was significantly decreased after 6 weeks of diets, from 83.4 at baseline to 37.3 (*P* < 0.001). In addition, we did not observe any significant correlation between FC level and microbiota diversity before and after the diets.

## Discussion

It has been hypothesized that a subset of patients with IBS may have an intolerance of dietary triggers such as gluten and highly FODMAP-containing foods, which can alter the gut microbiota and the metabolome of patients with IBS leading to worsening of their symptoms [21, 22]. This has led researchers to recommend LF-GFD as the most widely adopted diets to improve IBS symptoms [23, 24]. Despite the growing popularity of the GFD and low-FODMAP diet in patients with IBS, the beneficial impact of such dietary interventions on the restoration of intestinal microbiota dysbiosis has been lacking in this population.

In this 6-week, controlled, dietary intervention study, a clinically significant improvement in IBS-SSS was observed after the dietary intervention compared to the baseline. Several other clinical trials have also shown that a low-FODMAP diet associates with an improvement in IBS symptom scores and effectively increased the quality of life in patients with IBS [15, 22, 25–27]. It is now well documented that IBS is a condition in which several pathophysiological mechanisms are involved in its development and symptom severity. Among them, a distinct fecal microbiota composition and microbiome dysbiosis has been proposed as one of the key factors associated with the disease symptom severity [28–30].

There is increasing evidence that dietary interventions using low-FODMAP diets could improve functional gastrointestinal symptoms in IBS patients particularly through interactions with the gut microbiota [11, 15]. Furthermore, another study suggested that reduction of FODMAPs intake in a GFD consistently and significantly improved the gastrointestinal symptoms in IBS patients who were finally classified as non-celiac gluten sensitivity (NCGS) [31]. In our study, we found that the relative abundance of phylum Firmicutes was higher prior to the start of dietary interventions compared to the phylum Bacteroidetes in patients with IBS. However, there was a clear tendency to increased Bacteroidetes after the dietary interventions, and subsequently, the F/B ratio was significantly decreased. In a recent study by Dieterich et al., clinical and neurological symptoms of NCGS patients who consumed a low-FODMAP diet and especially the GFD significantly improved [32]. In addition, they reported a significant increase in the numbers of Bacteroidetes following a 2-week GFD compared to the low-FODMAP diet (*P* < 0.01). Furthermore, data obtained from a randomized clinical trial in childhood IBS demonstrated that individuals who respond to a low-FODMAP diet have a greater capacity for saccharolytic metabolism mainly due to higher proportions of Bacteroidaceae, Erysipilotrichaceae, and Clostridiales species than non-responders [33]. Rajilić-Stojanović et al. also reported an approximately twofold increase in the F/B ratio as the major bacterial phyla in 62 IBS patients (Rome II criteria) compared with 46 healthy subjects [34]. This finding has been observed in several other studies, in which the abundance of *Firmicutes* was enriched together with a reduced abundance of *Bacteroidetes* in the IBS subjects compared to healthy individuals [35, 36]. In contrast, other studies reported an increase in the content and abundance of *Bacteroidetes* members in the IBS patients compared to non-IBS subjects [37, 38]. *Bacteroidetes* are known as complex carbohydrate digesters which are specialized in degrading specific types of dietary fibers in order to maximize energy intake from these kinds of carbohydrates [39, 40].

Several studies have reported a significant depletion in *Actinobacteria* in the gut of patients with IBS [34, 36, 41]. On the other hand, other studies reported an increase in the relative abundance of Actinobacteria among IBS patients compared to healthy controls [35, 42, 43]. In our study the relative abundance of Actinobacteria was increased, although not statistically significant, after the LF-GFD intervention. In line with our results, McIntosh et al. also reported that a 3-week low-FODMAP diet increased Actinobacteria richness and diversity in patients with IBS [22]. In contrast, a recent study had demonstrated that gut bacteria such as Actinobacteria, *Bifidobacterium*, and *Faecalibacterium prausnitzii* were significantly decreased in IBS patients throughout a 9-week low-FODMAP diet along with a reduction in total SCFAs, n-butyric acid, and serum levels of proinflammatory cytokines (IL-6 and IL-8) as compared to baseline [44]. However, the overall inconsistency and differences in the abovementioned results contribute to the difference among study populations, IBS subtypes, duration of dietary intervention, level of dietary adherence, washout period in crossover studies, and variations in taste or other contents outside of FODMAP carbohydrates that may affect the dysbiotic gut microbiota and disease outcomes.

A number of studies have shown that the low-FOMAPs diet decreases the abundance of *Bifidobacterium* in the gut of IBS patients [12, 16, 22, 25, 27, 44]. Collectively, previous studies have also demonstrated that GFD induced a decrease in *Bifidobacterium* spp. in the intestinal microbiome of healthy human subjects, celiac disease (CD), and NCGS patients, raising potential concerns after the consumption of a GFD [32, 45, 46]. However, Collado et al. reported increased prevalence of certain *Bifidobacterium* species including *B. adolescentis*, *B. lactis*, and *B. dentium* after a GFD in CD subjects [47]. In another study from Brazil, the fecal counts of Bifidobacteria were significantly higher in GFD treated-CD (T-CD) patients compared to the healthy subjects [46, 48]. Interestingly, we observed that the relative abundances of lactate-producing bacteria *Bifidobacterium* and *Lactobacillus* were increased after the LF-GFD intervention in IBS patients. To our knowledge, no previous studies have evaluated the effects of the LF-GFD intervention simultaneously on IBS patients. Our findings propose that at least in patients with IBS implementing LF-GFD may lead to an increase in the relative abundances of *Bifidobacterium* and *Lactobacillus*, although contrary to the other studies which applied these dietary interventions separately.

The family Enterobacteriaceae (phylum Proteobacteria), contains several pathogenic genera such as *Escherichia*, *Shigella*, *Salmonella*, and *Campylobacter*. Generally, these aerobes were found to be slightly enriched in IBS patients and significantly correlated with IBS symptoms. Moreover, an increase in some of these pathogenic microbiota may contribute to the low rate of mucosal inflammation through overexpression of proinflammatory cytokines IL-6 and IL-8 as seen in IBS patients [29, 34, 49, 50]. In contrast, Tana et al. found no difference in Enterobacteriaceae count between IBS patients and healthy controls. In another study by De Palma et al., Enterobacteriaceae were increased in a group of healthy adult subjects who were on a one-month GFD [51]. In our study, we found no difference in the relative abundance of *Enterobacteriaceae* before and after the dietary intervention.

The genus *Streptococcus* is among the dominant bacterial groups present in the upper gastrointestinal tract [52]. Some reports have also showed high fecal amounts of *Streptococcus* spp., pathogenic bacteria which causes increased expression levels of IL-6 [53], in IBS patients [34], and particularly in IBS-D subtypes [54, 55]. We found no difference in the relative abundance of *Streptococcus* after the dietary intervention compared to the baseline. Our results are in agreement with another study that found no difference in the relative abundance of *Streptococcus* species for the low FODMAP diet compared with the sham diet [25]. Furthermore, Bennet et al. also demonstrated no difference in abundance of *Streptococcus* between responders and non-responders patients with IBS after a 4-week low-FODMAP diet [27].

In this study, over half of the patients had abnormal FC values before dietary intervention. Moreover, a major part of the patients were diagnosed to suffer from IBS-D subtype. It has been reported that alterations in some groups of gut microbiota may induce pro-inflammatory cytokines like IL-6 and IL-8, and thus such IBS patients probably suffer from a chronic low-grade inflammation [56]. In addition, a subgroup of IBS patients known as “IBS-D” may experience a higher level of inflammation [57, 58]. Therefore, alterations in certain groups of gut microbiota and the presence of IBS-D subtype can justify abnormal level of FC among such patients in our study.

Our data suggest that there is a correlation between consuming LF-GFD and decrease of FC which is in line with a study performed by Shulman et al. [59] but are in contrast with a few previous studies which suggested that FC concentration was not increased in IBS patients compared with control subjects [60, 61]. Therefore, it is noteworthy to administrate the diet or a combination of diets of choice for gut microbiota-associated disorders such as IBS in order to normalize the dysbiotic communities of microbiota. However, these controversial findings in the above-mentioned studies may be due to differences in the study design and population.

## Conclusions

In summary, our study suggests that patients with IBS who consumed LF-GFD had a significant improvement in IBS symptoms and normalization of their gut microbiota. In addition, our findings indicate inflammation in IBS patients. In which consuming LF-GFD can downregulate intestinal inflammation, and consequently decrease IBS-SSS. To the best of our knowledge, there has been no comprehensive analysis of the effects of LF-GFD on the gut microbiota in a group of unselected Iranian patients with IBS. We also suggest evaluating the effects of this combined dietary intervention on the metabolic output of gut microbiota and its integration with supplementary probiotics to avoid side effects on health due to the unfavorable alteration of the intestinal microbiota in IBS individuals. Future studies are still required to validate the robustness of our findings, and to establish a long-term efficacy and safety of this dietary intervention for personalized nutrition in IBS.

## Supplementary Information

**Additional file 1** Examples of high FODMAP gluten free foods and some examples of gluten-containing low FODMAP foods.

**Additional file 2** The taxon-specific primers used in this study.

**Additional file 3** Pie charts representing the mean percentage of the bacterial taxa that constitute the fecal microbiota in IBS patients before and after LFGFD.

## Data Availability

The datasets supporting the conclusions of this article are included within the article and its additional file.
