# Supplementary material for: Influence of low FODMAP-gluten free diet on gut microbiota alterations and symptom severity in Iranian patients with irritable bowel syndrome
Source: BMC Gastroenterol. 2021 Jul 14;21:292. doi: 10.1186/s12876-021-01868-5 (PMC8278734; doi:10.1186/s12876-021-01868-5)
Supplement: Supplementary file 1 — Additional file 1 Examples of high FODMAP gluten free foods and some examples of gluten-containing low FODMAP foods. [file 12876_2021_1868_MOESM1_ESM.docx]

**Table S1.** Examples of high FODMAP gluten free foods and some examples of gluten-containing low FODMAP foods.

| **High FODMAP gluten-free foods** | **Gluten-containing low FODMAP foods** |
| --- | --- |
| Soy flour | Soy sauce (2 tablespoons) |
| Coconut flour | Wheat-based snacks such as 2 small plain biscuits |
| Chickpea flour | Sourdough (spelt or wheat) bread |
| Dried fruits including dates, mango, sultanas and pear | Low FODMAP products with tiny amounts of gluten such as yeast spread (Vegemite) |
| Various fresh fruit and vegetables including onion and garlic | Also gluten cross-contamination of low FODMAP foods |
| Honey |  |
| Inulin and chicory root |  |
