# Supplementary material for: Influence of low FODMAP-gluten free diet on gut microbiota alterations and symptom severity in Iranian patients with irritable bowel syndrome
Source: BMC Gastroenterol. 2021 Jul 14;21:292. doi: 10.1186/s12876-021-01868-5 (PMC8278734; doi:10.1186/s12876-021-01868-5)
Supplement: Supplementary file 2 — Additional file 2 The taxon-specific primers used in this study. [file 12876_2021_1868_MOESM2_ESM.docx]

**Table S2.** The taxon-specific primers used in this study.

| **Target taxon** | **Primer name** | **Primer sequence (5ꞌ-3ꞌ)** | **Amplicon length (bp)** | **Reference** |
| --- | --- | --- | --- | --- |
| Eubacteria | UniF340  UniR514 | ACTCCTACGGGAGGCAGCAGT  ATTACCGCGGCTGCTGGC | ~ 200 bp | [1] |
| Lactobacillus spp. | Lacto-F  Lacto-R | TGGATGCCTTGGCACTAG  AAATCTCCGGATCAAAGCTTAC | ~ 89 bp | [2] |
| Bifidobacterium spp. | Bifid-F  Bifid-R | GGGATGCTGGTGTGGAAGAG  TGCTCGCGTCCACTATCCAG | ~ 200 bp | [2] |
| Bacteroidetes | Bac960-F  Bac1100-R | GTTTAATTCGATGATACGCG  TTAAGCCGACACCTCACG | ~ 137 bp | [3] |
| Firmicutes | Firm934-F  Firm1060-R | GGAG**Y**ATGTGGTTTAATTCGAAGCA  AGCTGACGACAACCATGCAC | ~ 129 bp | [3] |
| Actinobacteria | Actino-F  Actino-R | GCG**K**CCTATCAGCTTGTTGGTG  CCGCCTACGAGC**Y**CTTTACGC | ~ 333 bp | [4] |
| Enterobacteriaceae | Enterob-F  Enterob-R | CGTCGCAAGM**M**CAAAGAG  TTACCGCGGCTGCTGGCAC | ~ 351 bp | [4] |
| Streptococcus spp. | Str1-F  Str2-R | GTACAGTTGCTTCAGGACGT  GTTCGATTTCRTCACGTTG | ~ 195 bp | [2] |
| Ruminococcus | Rflbr730F  Clep866mR | GGCGGCYT**R**CTGGGCTTT  GCAGGTGGAT**W**ACTTATTGTGTTAA | ~ 157 bp | [5] |

The nucleotides in bold type represent: Y, C or T; K, G or T; M, A or C; R, A or G; W, A or T.

**References:**

1. Moraes JG, Motta ME, Beltrão MF, Salviano TL, Silva GA. Fecal microbiota and diet of children with chronic constipation. International journal of pediatrics. 2016 Jun 23;2016.
2. Wang IK, Lai HC, Yu CJ, Liang CC, Chang CT, Kuo HL, Yang YF, Lin CC, Lin HH, Liu YL, Chang YC. Real-time PCR analysis of the intestinal microbiotas in peritoneal dialysis patients. Applied and environmental microbiology. 2012 Feb 15;78(4):1107-12.
3. Matsuki T, Watanabe K, Fujimoto J, Takada T, Tanaka R. Use of 16S rRNA gene-targeted group-specific primers for real-time PCR analysis of predominant bacteria in human feces. Applied and environmental microbiology. 2004 Dec 1;70(12):7220-8.
4. Hermann-Bank ML, Skovgaard K, Stockmarr A, Larsen N, Mølbak L. The Gut Microbiotassay: a high-throughput qPCR approach combinable with next generation sequencing to study gut microbial diversity. BMC genomics. 2013 Dec;14(1):1-4.
5. Ahmed SM, El-Hefnawy AM, Azouz H, Meheissen MA, Hamdy M, Roshdy YS, Ibrahim AE. Study of the Gut Enterotypes in Egyptian Children with Autism Spectrum Disorder. Microbiology Research Journal International. 2018 May 17:1-9.
