# Supplementary material for: Influence of low FODMAP-gluten free diet on gut microbiota alterations and symptom severity in Iranian patients with irritable bowel syndrome
Source: BMC Gastroenterol. 2021 Jul 14;21:292. doi: 10.1186/s12876-021-01868-5 (PMC8278734; doi:10.1186/s12876-021-01868-5)
Supplement: Supplementary file 3 — Additional file 3 Pie charts representing the mean percentage of the bacterial taxa that constitute the fecal microbiota in IBS patients before and after LFGFD. [file 12876_2021_1868_MOESM3_ESM.docx]

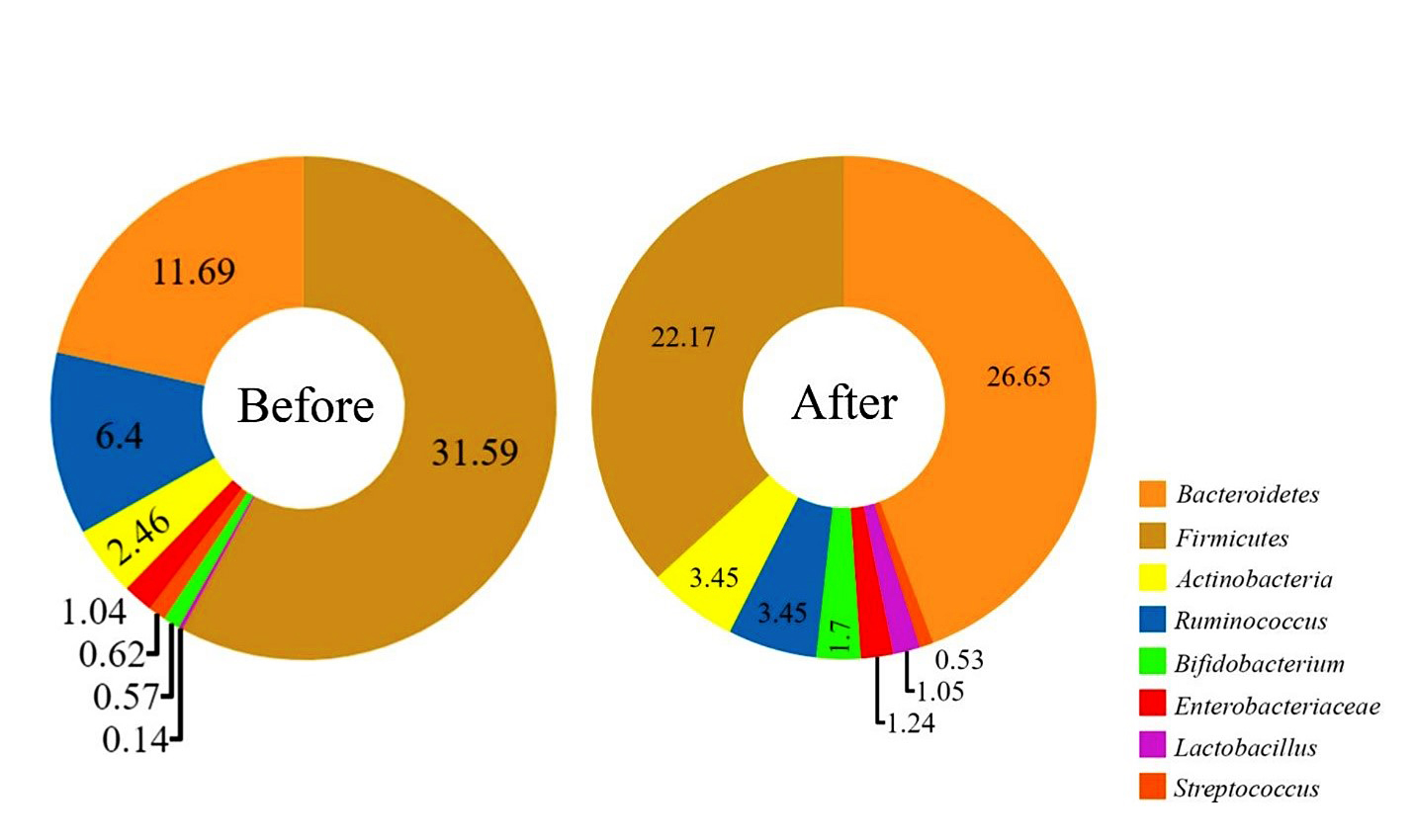


**Figure S1**. Pie charts representing the mean percentage of the bacterial taxa that constitute the fecal microbiota in IBS patients before and after LF-GFD.
